# Sarcopenia and 24-Month All-Cause Mortality in Patients Receiving Maintenance Hemodialysis: A Prospective Observational Cohort Study Applying the Revised European Working Group on Sarcopenia in Older People Criteria

**DOI:** 10.3390/medicina62091735

**Published:** 2026-09-09

**Authors:** Zorica M. Dimitrijevic, Jelena Randjelovic, Danijela Tasic, Karolina Paunovic, Branislav Apostolovic, Emina Kostic, Tamara Vrecic, Aleksandar Radivojevic, Branka Mitic

**Affiliations:** 1 Clinic for Nephrology, University Clinical Center Nis, Bulevar dr Zorana Djindjica 48, 18000 Nis, Serbia; jelenastole@yahoo.com (J.R.); danijeladt@gmail.com (D.T.); paunovickarolina@gmail.com (K.P.); bane.apostolovic@gmail.com (B.A.); kosticemina2@yahoo.com (E.K.); tamaravrecic@live.com (T.V.); x.arnef199@gmail.com (A.R.); dmmitic@ptt.rs (B.M.); 2 Faculty of Medicine, University of Nis, Bulevar dr Zorana Djindjica 81, 18000 Nis, Serbia

**Keywords:** handgrip strength, appendicular skeletal muscle mass index, bioelectrical impedance analysis, end-stage kidney disease, muscle strength, protein-energy wasting

## Abstract

*Background and Objectives*: Sarcopenia is common among patients receiving maintenance hemodialysis, although prevalence estimates vary according to the diagnostic approach. We assessed sarcopenia defined according to the revised European Working Group on Sarcopenia in Older People (EWGSOP2) criteria, its baseline correlates, and its association with 24-month all-cause mortality. *Materials and Methods*: In this single-center prospective cohort at a tertiary nephrology center, 176 patients enrolled in December 2022 were followed for 24 months. Confirmed sarcopenia required both low handgrip strength and a low appendicular skeletal muscle index assessed by bioelectrical impedance. Physical performance was not assessed. Because the proportional-hazards assumption was not satisfied, restricted mean survival time (RMST) was used as the primary measure of the survival difference. *Results*: Sarcopenia was present in 46 of 176 participants (26.1%, 95% CI 20.2–33.1). Older age, lower serum albumin and phosphorus concentrations, lower Kt/V, and longer time on maintenance hemodialysis were independently associated with sarcopenia. Thirty-one participants died during follow-up, and survival was lower among those with sarcopenia (log-rank *p* = 0.035). The adjusted RMST difference was −3.88 months (95% CI −6.49 to −1.28; *p* = 0.003). A Cox sensitivity analysis yielded a hazard ratio of 2.62 (95% CI 1.06–6.45). There was no evidence of a sarcopenia-by-sex interaction in the adjusted RMST analysis (*p* = 0.964). *Conclusions*: Confirmed sarcopenia was present in approximately one quarter of patients and was associated with approximately four months shorter restricted mean survival over 24 months. EWGSOP2-based assessment may provide prognostic information in this setting; whether routine screening or management based on sarcopenia status improves clinical outcomes remains to be determined.

## 1. Introduction

Sarcopenia is a progressive and generalized skeletal muscle disorder characterized by loss of muscle strength and muscle mass. It is associated with impaired physical function, falls, fractures, prolonged hospitalization, loss of independence, and premature death, and has become an increasingly important public health concern with population aging [1,2,3]. The revised consensus of the European Working Group on Sarcopenia in Older People (EWGSOP2) placed low muscle strength at the beginning of the diagnostic algorithm, with low muscle quantity required to confirm the diagnosis and impaired physical performance used to grade severity [1]. This represented an important change from the 2010 definition, in which low muscle mass was the entry criterion [4]. The revised approach reflects evidence that muscle strength declines earlier and more rapidly than muscle mass, is more consistently associated with adverse outcomes, and can be assessed relatively easily in clinical practice. The two algorithms therefore do not necessarily identify the same patients, and prevalence or effect estimates obtained using one definition cannot be assumed to apply directly to the other.

Patients with chronic kidney disease, particularly those receiving maintenance hemodialysis, are exposed to several factors that may contribute to muscle loss. Reduced physical activity and prolonged inactivity associated with dialysis sessions, chronic low-grade inflammation, metabolic acidosis, insulin resistance and other hormonal disturbances, inadequate nutrient intake, and accelerated muscle protein catabolism may act together within the uremic milieu [5,6,7,8,9,10]. The resulting phenotype overlaps with, but is not identical to, protein-energy wasting, which is defined by concurrent biochemical, body-mass, and muscle-mass criteria in kidney disease [11]. Muscle impairment in this population is not limited to reduced quantity. Magnetic resonance imaging studies in patients receiving hemodialysis have demonstrated lower-limb muscle atrophy together with increased intramuscular non-contractile tissue, indicating that gross cross-sectional area may not fully reflect the amount of contractile tissue available; the degree of atrophy has also been related to physical performance [12]. Muscle-mass measurements alone may therefore provide an incomplete description of clinically relevant muscle impairment, supporting an approach in which muscle strength is assessed early in the diagnostic pathway.

Assessment of sarcopenia in patients receiving hemodialysis is methodologically challenging. Body composition varies across the dialysis cycle, and interdialytic fluid accumulation alters the relationship between intracellular and extracellular water on which impedance-based estimates of muscle mass depend. The timing of measurement relative to the dialysis session can therefore influence the result. In addition, most reference equations and diagnostic thresholds were derived from community-dwelling older adults rather than from patients with kidney failure, and kidney-specific consensus cut-offs are not currently available [13]. The method used to assess muscle mass may also influence classification; in hemodialysis cohorts, the diagnosis of sarcopenia has been shown to depend substantially on the muscle-mass component and therefore on the method used to estimate it [14]. For this reason, prevalence estimates should be interpreted in the context of the diagnostic algorithm, measurement method, and timing of assessment.

Sarcopenia has been consistently associated with increased mortality in patients receiving dialysis, both in systematic reviews with meta-analysis and in individual cohort studies [15,16,17,18]. However, several questions remain. First, much of the available evidence is based on heterogeneous definitions, and many studies have used muscle mass alone or individual components of the sarcopenia phenotype. It is therefore less clear how consistently the association is observed when the integrated EWGSOP2 algorithm, requiring both low strength and low muscle mass, is applied [14,15]. Because the strength-first algorithm identifies a different and generally smaller group of patients than mass-based definitions, estimates obtained using earlier criteria cannot be directly transferred to EWGSOP2-defined sarcopenia. Second, most published dialysis cohorts originate from East Asian or Western European populations, whereas data from South-eastern European hemodialysis populations remain limited. Third, the clinical, nutritional, and dialysis-related characteristics accompanying EWGSOP2-defined sarcopenia in this population, as well as possible differences in its prognostic association between men and women, remain incompletely characterized. Finally, most published survival estimates are reported as hazard ratios, while the proportional-hazards assumption is not consistently tested or reported and may not always be satisfied.

Accordingly, this study aimed to (1) estimate the prevalence of confirmed sarcopenia according to EWGSOP2 criteria in a cohort of patients receiving maintenance hemodialysis at a single tertiary center; (2) identify clinical and biochemical factors associated with sarcopenia at baseline; and (3) examine the association between baseline sarcopenia and 24-month all-cause mortality. We hypothesized that sarcopenia would be associated with poorer survival after adjustment for demographic and dialysis-related characteristics. Because sarcopenia and its baseline correlates were assessed at the same time point, the second aim was descriptive and was not intended to support causal or temporal inference.

## 2. Materials and Methods

### 2.1. Study Design, Setting and Participants

This was a single-center prospective observational cohort study combining a baseline cross-sectional assessment of sarcopenia with prospective 24-month follow-up for all-cause mortality. The study is reported in accordance with the Strengthening the Reporting of Observational Studies in Epidemiology (STROBE) statement [19].

The study was conducted at the Clinic for Nephrology, University Clinical Center Nis, Serbia, a tertiary-care university nephrology center. Baseline assessments, comprising handgrip strength measurement and bioelectrical impedance analysis, were performed between 15 December 2022 and 30 December 2022. Follow-up for vital status extended to 3 January 2025, completing 24 months for every participant.

All patients receiving maintenance hemodialysis at the center during the baseline period were assessed for eligibility; participants were enrolled consecutively, and no sampling procedure was applied. Patients were eligible if they were aged 18 years or older, had been receiving maintenance hemodialysis for more than three months through a functioning arteriovenous fistula, were clinically stable, and were able to complete the assessments required for classification of sarcopenia.

Patients were excluded if any of the following applied at the time of the planned baseline assessment: an implanted cardiac pacemaker, because of interference with bioelectrical impedance measurement; lower-limb amputation, because appendicular skeletal muscle mass cannot be validly estimated by impedance in the presence of a missing limb; marked interdialytic weight gain exceeding 5 kg, because the associated fluid excess would have substantially biased impedance-derived estimates of muscle mass; and inability to perform the handgrip-strength test reliably despite instruction, whether because of cognitive limitation, motor impairment of both upper limbs, or inability to follow the standardized testing procedure.

**Figure 1:**
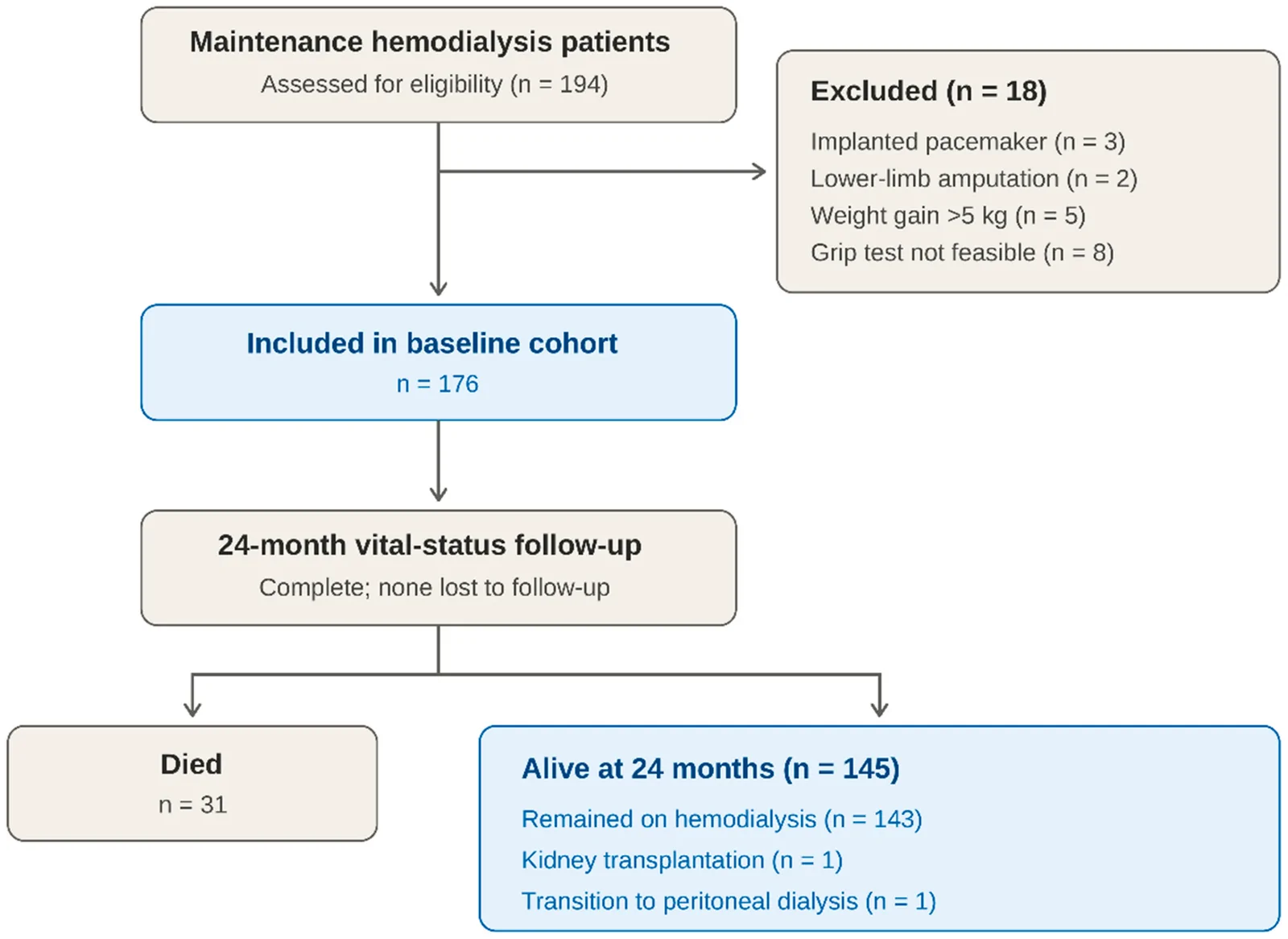
Flow diagram of participant eligibility assessment, exclusions, inclusion and 24-month follow-up.

A total of 194 patients were assessed for eligibility, and 18 were excluded: 3 because of an implanted cardiac pacemaker, 2 because of lower-limb amputation, 5 because of marked interdialytic weight gain exceeding 5 kg, and 8 because they were unable to perform the handgrip-strength test reliably. The analyzed cohort therefore comprised 176 participants. Screening, exclusions, and 24-month follow-up are summarized in Figure 1.

No a priori sample-size calculation was performed. The study was designed to include the entire eligible prevalent hemodialysis population treated at the center during the baseline period, and the sample size was therefore determined by the size of that population rather than by a target precision. The implications of this for statistical power, particularly for subgroup and interaction analyses, are addressed in the Discussion.

The study was conducted in accordance with the Declaration of Helsinki and was approved by the Ethics Committee of the University Clinical Center Nis, Serbia (approval number 35828, 27 November 2022). All participants provided written informed consent before any study procedure.

### 2.2. Clinical, Dialysis and Laboratory Assessment

Baseline variables were age, sex, primary cause of end-stage kidney disease (ESKD), time on maintenance hemodialysis, dialysis adequacy, and anthropometry. Time on maintenance hemodialysis was recorded in years from the first maintenance hemodialysis session. Dialysis adequacy was expressed as single-pool Kt/V calculated by the Daugirdas second-generation formula [20] from the mid-week session and was analyzed as a continuous variable; no threshold value of Kt/V was used in any analysis.

Body weight was measured after the dialysis session on a calibrated digital scale with participants in light clothing and without shoes; height was measured with a stadiometer, and waist circumference was measured at the midpoint between the lowest rib and the iliac crest at the end of normal expiration. Body mass index was calculated as weight divided by height squared.

Blood was sampled before the mid-week hemodialysis session and before administration of intradialytic anticoagulation, using the same standardized schedule for all participants. Serum biochemistry was measured on an automated clinical chemistry analyzer (Dimension RxL Max Integrated Chemistry System, Siemens Healthcare Diagnostics, Erlangen, Germany).

### 2.3. Assessment of Sarcopenia

Sarcopenia was assessed according to the EWGSOP2 criteria [1]. Confirmed sarcopenia was defined as the presence of both low muscle strength and low muscle mass. Physical performance was not assessed because baseline measurements were performed within the routine workflow of the dialysis unit. Consequently, sarcopenia severity could not be graded, and severe sarcopenia was not classified. The implications of this omission for the functional characterization of the cohort are considered in the Discussion.

Handgrip strength was measured in kilograms using a Jamar Hydraulic Hand Dynamometer (Jamar, Performance Health, Downers Grove, IL, USA, measurement range 0–90 kg [0–200 lb]), with the handle set at the second position. The device was supplied with a calibration certificate (serial number 202507102051) documenting calibration on 10 August 2022 using 12 reference loads across all five handle positions; the next calibration was due on 10 August 2023. All baseline handgrip-strength measurements were performed in December 2022, within this documented calibration interval. Participants were positioned in a standardized manner: seated, shoulder adducted and neutrally rotated, elbow flexed at 90°, forearm in neutral rotation, and wrist between 0° and 30° of dorsiflexion. Testing was performed on the arm without the arteriovenous fistula to ensure that hand dominance did not influence the side tested; hand dominance was not recorded. A submaximal familiarization trial was conducted before formal testing. Three maximal voluntary efforts were performed with standardized verbal encouragement and approximately one minute between efforts; the highest value was used, consistent with EWGSOP2 recommendations. Low muscle strength was defined as handgrip strength below 27 kg in men and below 16 kg in women [1].

Muscle mass. Body composition was assessed by multifrequency bioelectrical impedance analysis (5–1000 kHz) using the Body Composition Monitor (Fresenius Medical Care, Bad Homburg, Germany), with measurements performed immediately after the mid-week hemodialysis session, when participants were closest to their prescribed post-dialysis weight, in order to limit the influence of extracellular fluid excess on impedance-derived estimates. Appendicular skeletal muscle mass was estimated using the Sergi equation [21] and indexed to height squared to yield the appendicular skeletal muscle index (ASMI). Low muscle mass was defined as ASMI below 7.0 kg/m^2^ in men and below 5.5 kg/m^2^ in women [1]. No study-specific or data-derived cut-off values were applied. The Sergi equation was developed and cross-validated in free-living Caucasian older adults rather than in patients with ESKD, in whom hydration coefficients and tissue composition differ; this is addressed as a limitation.

### 2.4. Potential Confounders, Effect Modifiers and Control of Bias

Covariates were selected on the basis of established clinical relevance to muscle wasting and mortality in kidney failure rather than on the basis of statistical significance in the present dataset. For the analysis of factors associated with sarcopenia, the multivariable model included age, sex, serum albumin, serum phosphorus, Kt/V, and time on maintenance hemodialysis. Diabetes mellitus was additionally examined in univariable analysis because of its recognized relationship with muscle loss; in this cohort, all participants with diabetes had diabetic kidney disease as the cause of ESKD, so the etiological category and the presence of diabetes coincide. Univariable estimates are reported for all examined variables, whereas multivariable estimates are reported for the six-variable prespecified model. No automated forward, backward, or stepwise variable-selection procedure was used.

Several clinically relevant characteristics were not part of the predefined dataset and could not be incorporated retrospectively: cardiovascular comorbidity burden and formal comorbidity indices, frailty, physical activity, dietary protein intake, normalized protein catabolic rate, hospitalization burden, and residual kidney function. C-reactive protein was available as a single baseline measurement and was not treated as a comprehensive assessment of inflammatory burden. Composite nutritional risk indices could not be derived because total cholesterol and total lymphocyte count were not recorded. The resulting potential for residual confounding is discussed explicitly in Section 4.

For the primary adjusted survival analysis, the covariate set comprised age, sex, and time on maintenance hemodialysis. The same adjustment set was used for the primary adjusted RMST analysis and the Cox sensitivity model. A further Cox sensitivity model additionally included serum albumin and serum phosphorus to examine whether the association between sarcopenia and mortality was materially altered after accounting for these clinically relevant nutritional and biochemical variables. Kt/V was examined in a separate sensitivity analysis by adding it to the primary adjustment set. Because only 31 deaths occurred, the number of covariates in the survival models was deliberately limited to reduce the risk of overfitting. The primary Cox model contained four parameters, including sarcopenia, corresponding to approximately 7.8 events per parameter, whereas the extended model including serum albumin and serum phosphorus contained six parameters, corresponding to approximately 5.2 events per parameter. The limited number of events and consequent risk of model instability are acknowledged as study limitations.

Sex was specified as the potential effect modifier of primary interest and was evaluated using a formal sarcopenia-by-sex interaction term rather than by comparing statistical significance within sex-specific strata. Sex-stratified survival analyses were therefore considered exploratory.

Several measures were applied to limit measurement bias. Handgrip strength was measured with the same device and an identical protocol in all participants by one trained investigator. Bioimpedance measurements were standardized to the post-dialysis period of the mid-week session. Laboratory sampling followed an identical pre-dialysis schedule for all participants. Vital status was ascertained for every participant, eliminating attrition bias for the primary outcome. Investigators performing baseline measurements were not involved in outcome ascertainment.

### 2.5. Outcome and Follow-Up

The outcome was all-cause mortality within 24 months of baseline assessment. Deaths were identified from the hospital electronic medical record and the dialysis unit register. Cause-specific mortality was not adjudicated.

Vital status was followed irrespective of subsequent change in renal replacement modality. During follow-up, one participant underwent kidney transplantation and one transferred to peritoneal dialysis; these participants remained under observation for vital status and were not censored at the time of modality change. Participants alive at the end of the observation period were administratively censored at 24 months.

### 2.6. Missing Data

Complete data were available for all variables in all 176 participants; no imputation was required, and no participant was excluded from any analysis because of missing data.

### 2.7. Statistical Analysis

Statistical analyses were performed using IBM SPSS Statistics, version 22.0 (IBM Corp., Armonk, NY, USA). Survival diagnostics and restricted survival-time analyses were performed in R, version 4.5.3 (R Foundation for Statistical Computing, Vienna, Austria), using the survival, survRM2, sandwich, and lmtest packages. Distributional characteristics of continuous variables were examined using the Shapiro–Wilk test together with visual inspection of histograms and quantile–quantile plots. Approximately normally distributed variables are presented as mean ± standard deviation, skewed variables as median with interquartile range, and categorical variables as number and percentage. Baseline characteristics are presented descriptively without hypothesis testing. The prevalence of sarcopenia is reported with a 95% confidence interval calculated using the Wilson score method. Complete data were available for all variables included in the analyses; therefore, no imputation was required.

Associations between baseline characteristics and sarcopenia were examined using univariable logistic regression. Candidate variables prespecified in Section 2.4 were entered simultaneously into a single multivariable logistic regression model. Continuous predictors were retained as continuous variables and were not categorized. To facilitate clinical interpretation, serum phosphorus and Kt/V were expressed per 0.1 mmol/L and per 0.1-unit increment, respectively. Estimates are reported as odds ratios (ORs) with 95% confidence intervals (CIs).

Survival was described using Kaplan–Meier curves and compared between participants with and without sarcopenia using the log-rank test. The proportional-hazards assumption was assessed using scaled Schoenfeld residuals and was additionally evaluated by including a time-dependent interaction between sarcopenia and the logarithm of survival time in the Cox model. Because the proportional-hazards assumption was not supported for sarcopenia, restricted mean survival time (RMST) over the prespecified 24-month follow-up period was used as the primary measure of the survival difference.

RMST was defined as the area under the Kaplan–Meier survival curve from baseline to 24 months. Because no participant was censored before 24 months, restricted survival time was completely observed for all participants and corresponded to the time to death for participants who died and 24 months for those who remained alive. Adjusted differences in restricted survival time were estimated using multivariable linear regression with heteroscedasticity-robust HC3 standard errors, adjusting for age, sex, and time on maintenance hemodialysis. Covariate-adjusted group-specific restricted mean survival times were obtained by marginal standardization from the fitted model.

A Cox regression model adjusted for the same covariates was performed as a sensitivity analysis. Because the proportional-hazards assumption was not satisfied for sarcopenia, the Cox hazard ratio was considered a sensitivity summary measure and was not interpreted as a constant relative hazard throughout follow-up. Effect modification by sex was assessed by including a sarcopenia-by-sex interaction term in both the restricted survival-time regression model and the Cox sensitivity model. An additional sensitivity analysis added Kt/V to both adjusted survival models. All tests were two-sided, and *p* < 0.05 was considered statistically significant.

Analyses of baseline factors associated with sarcopenia were considered secondary, whereas sex-stratified analyses and additional sensitivity analyses were regarded as exploratory. No adjustment for multiple comparisons was applied to these secondary and exploratory analyses, and the potential inflation of the type I error rate was considered when interpreting the findings.

## 3. Results

### 3.1. Participant Flow

A total of 194 patients receiving maintenance hemodialysis were assessed for eligibility, of whom 18 were excluded before baseline assessment for the reasons and numbers shown in Figure 1, leaving 176 participants in the analyzed cohort. During 24 months of follow-up, 31 participants died, and 145 were alive at the end of the observation period; of the survivors, 143 remained on maintenance hemodialysis, 1 underwent kidney transplantation, and 1 transitioned to peritoneal dialysis. Vital status was ascertained for every participant, and no participant was lost to follow-up.

Exclusion criteria were applied at the time of the planned baseline assessment. Weight gain refers to interdialytic weight gain exceeding 5 kg. Grip test not feasible denotes inability to perform the handgrip-strength test reliably despite instruction. Participants who underwent kidney transplantation or transitioned to peritoneal dialysis remained under observation for vital status and were not censored at the time of modality change. HD, hemodialysis.

### 3.2. Baseline Characteristics

The cohort comprised 106 men (60.2%) and 70 women (39.8%), with a mean age of 57.5 ± 13.0 years. The most frequent primary cause of ESKD was hypertension, followed by other or unknown causes and diabetes. Baseline demographic, dialysis-related, anthropometric, and laboratory characteristics are shown in Table 1. As expected from established physiological differences, men had higher body weight, height, waist circumference, ASMI, and handgrip strength, and women had a higher body fat percentage; these differences were not formally tested, as sex comparisons were not a study objective.

### 3.3. Prevalence of Sarcopenia and Characteristics by Sarcopenia Status

Confirmed sarcopenia was identified in 46 of 176 participants, a prevalence of 26.1% (95% CI 20.2–33.1). Sarcopenia was present in 26 of 106 men (24.5%) and 20 of 70 women (28.6%). Baseline characteristics by sex and sarcopenia status are presented descriptively in Table 2. Participants with sarcopenia were older, had spent longer on maintenance hemodialysis, had lower Kt/V, lower body weight, body mass index, and waist circumference, higher body fat percentage, lower serum albumin, calcium, and phosphorus, and higher C-reactive protein. As follows directly from the diagnostic definition, ASMI and handgrip strength were lower in participants with sarcopenia.

**Table 2:** Baseline clinical and biochemical characteristics by sex and sarcopenia status.

Parameter	Men, No Sarcopenia (n = 80)	Men, Sarcopenia (n = 26)	Women, No Sarcopenia (n = 50)	Women, Sarcopenia (n = 20)
Age, years	55.2 ± 13.4	64.0 ± 10.3	54.6 ± 12.6	65.3 ± 9.3
Time on maintenance hemodialysis, years	5.0 ± 1.7	7.2 ± 1.3	5.2 ± 1.9	7.6 ± 1.8
Kt/V	1.34 ± 0.36	1.20 ± 0.12	1.41 ± 0.29	1.19 ± 0.28
Weight, kg	74.3 ± 12.8	69.2 ± 10.2	59.3 ± 15.3	50.5 ± 8.9
Body mass index, kg/m^2^	26.1 ± 6.1	22.4 ± 4.3	25.3 ± 5.6	22.8 ± 3.1
Waist circumference, cm	95.2 ± 10.2	89.3 ± 11.3	91.4 ± 8.2	78.3 ± 11.1
Body fat, %	22.2 ± 5.4	32.6 ± 8.4	32.4 ± 6.3	38.4 ± 7.3
ASMI, kg/m^2^	7.7 ± 1.0	5.8 ± 0.9	6.4 ± 0.9	5.0 ± 0.4
Handgrip strength, kg	26.7 ± 7.4	18.3 ± 4.3	20.5 ± 4.1	15.2 ± 2.2
Hemoglobin, g/dL	11.0 ± 2.3	10.7 ± 1.2	10.5 ± 0.6	10.3 ± 1.1
Albumin, g/L	38.1 ± 3.8	34.3 ± 5.1	35.7 ± 2.7	33.6 ± 1.7
C-reactive protein, mg/L	8.5 [7.8–9.4]	10.2 [8.9–12.7]	8.9 [7.5–9.9]	11.2 [9.5–14.1]
Creatinine, µmol/L	569.0 ± 381.3	608.8 ± 255.2	588.4 ± 197.6	487.1 ± 288.3
Calcium, mmol/L	2.30 ± 0.07	2.08 ± 0.14	2.29 ± 0.40	2.16 ± 0.20
Phosphorus, mmol/L	1.56 ± 0.30	1.21 ± 0.35	1.59 ± 0.36	1.15 ± 0.30
Parathyroid hormone, pg/mL	556.0 ± 358.2	439.0 ± 396.2	426.0 ± 255.3	417.0 ± 284.2

Data are mean ± standard deviation or median [interquartile range]. Baseline characteristics are presented descriptively; formal comparisons between groups are provided by the regression analyses in Table 3. ASMI, appendicular skeletal muscle index; Kt/V, single-pool dialysis adequacy index.

**Table 3:** Univariable and multivariable logistic regression analyses of factors associated with sarcopenia (n = 176).

Variable	Univariable OR (95% CI)	*p*	Adjusted OR (95% CI)	*p*
Age, per year	1.07 (1.03–1.10)	<0.001	1.08 (1.02–1.14)	0.005
Female sex	1.23 (0.62–2.43)	0.550	0.37 (0.10–1.34)	0.130
Serum albumin, per 1 g/L	0.79 (0.71–0.88)	<0.001	0.71 (0.60–0.86)	<0.001
Serum phosphorus, per 0.1 mmol/L	0.71 (0.63–0.80)	<0.001	0.63 (0.51–0.76)	<0.001
Kt/V, per 0.1 unit	0.82 (0.73–0.93)	0.002	0.74 (0.58–0.94)	0.015
Time on maintenance hemodialysis, per year	2.21 (1.68–2.90)	<0.001	2.60 (1.71–3.95)	<0.001
Diabetes mellitus (all with diabetic kidney disease)	1.56 (0.71–3.45)	0.273	—	—

CI, confidence interval; ESKD, end-stage kidney disease; OR, odds ratio. The multivariable model included age, sex, serum albumin, serum phosphorus, Kt/V, and time on maintenance hemodialysis, all entered simultaneously; no stepwise selection was applied. Serum phosphorus and Kt/V are expressed per 0.1-unit increment to aid interpretation. All participants with diabetes had diabetic kidney disease as the cause of ESKD; diabetes was examined in univariable analysis only.

### 3.4. Factors Associated with Sarcopenia

Univariable and multivariable logistic regression results are shown in Table 3. In the multivariable model, older age, lower serum albumin, lower serum phosphorus, lower Kt/V, and longer time on maintenance hemodialysis remained associated with sarcopenia. Female sex was not independently associated with sarcopenia. Diabetes mellitus, which in this cohort was present only in participants with diabetic kidney disease as the cause of ESKD, was not associated with sarcopenia in univariable analysis (Table 3).

### 3.5. Twenty-Four-Month All-Cause Mortality

**Figure 2:**
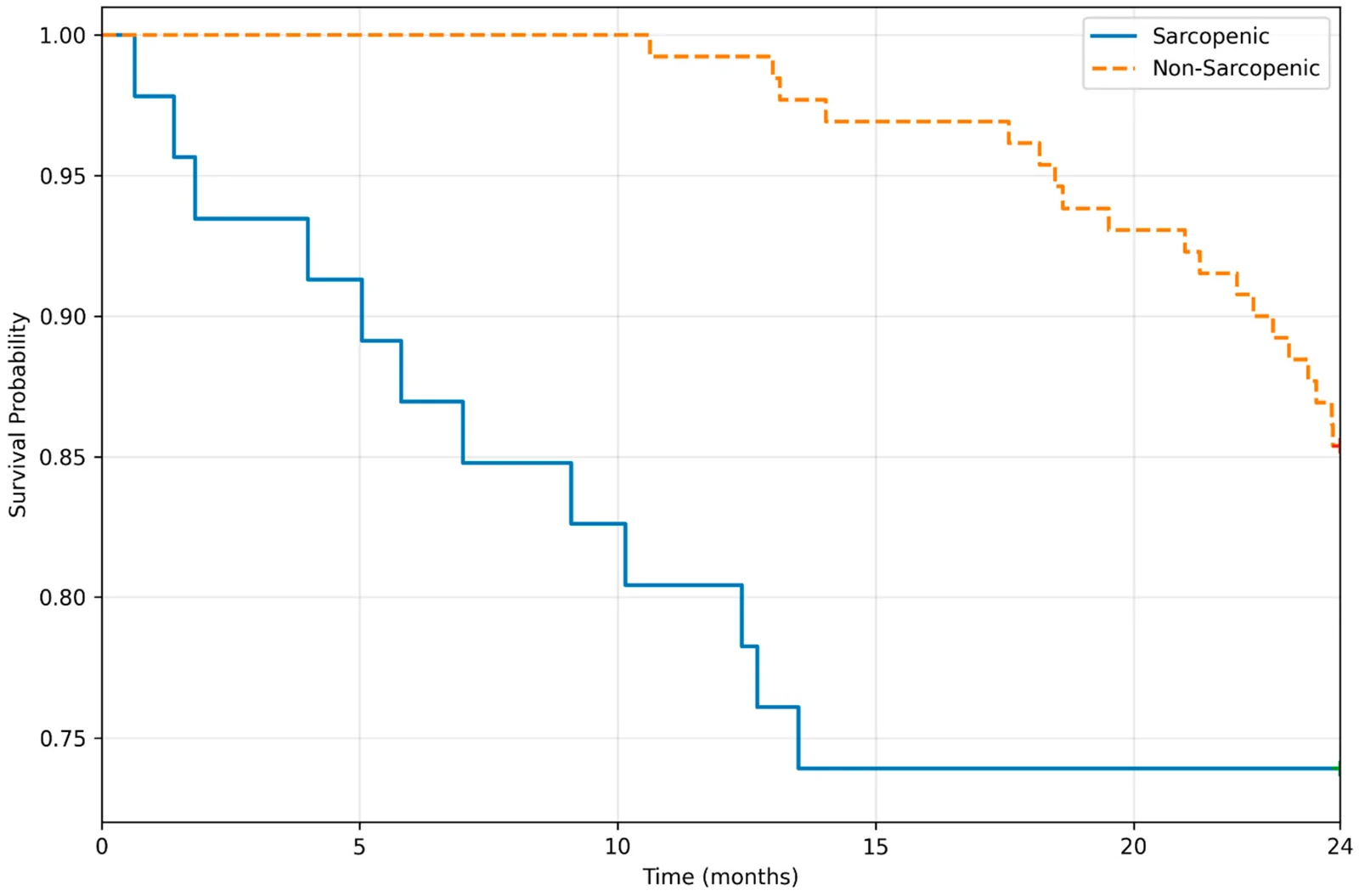
Kaplan–Meier survival curves for 24-month all-cause mortality in patients receiving maintenance hemodialysis, with and without sarcopenia. Survival was lower among participants with sarcopenia (log-rank *p* = 0.035).

During follow-up, 31 of 176 participants died. Death occurred in 12 of 46 participants with sarcopenia (26.1%) and in 19 of 130 participants without sarcopenia (14.6%). Kaplan–Meier analysis demonstrated lower survival among participants with sarcopenia (log-rank χ^2^ = 4.47, *p* = 0.035; Figure 2).

The proportional-hazards assumption was not satisfied for sarcopenia: the time-dependent interaction between sarcopenia and the logarithm of survival time was statistically significant (*p* = 0.036), and the scaled Schoenfeld residual test was significant both for sarcopenia (*p* < 0.001) and for the model globally (*p* < 0.001). RMST over 24 months was therefore used as the primary measure of the survival difference, and the Cox models reported below were considered sensitivity analyses.

The unadjusted 24-month RMST was 19.56 months among participants with sarcopenia and 23.34 months among those without sarcopenia. This corresponded to 3.78 fewer months of survival among participants with sarcopenia (95% CI −6.12 to −1.44; *p* = 0.002). After adjustment for age, sex, and time on maintenance hemodialysis, the standardized RMST estimates were 19.48 months for participants with sarcopenia and 23.37 months for those without sarcopenia, corresponding to an adjusted RMST difference of −3.88 months (95% CI −6.49 to −1.28; *p* = 0.003) (Table 4).

**Table 4:** Restricted mean survival time according to sarcopenia status over 24 months.

Analysis	No Sarcopenia	Sarcopenia	RMST Difference (95% CI)	*p*-Value
Unadjusted RMST, months	23.34	19.56	−3.78 (−6.12 to −1.44)	0.002
Adjusted RMST, months *	23.37	19.48	−3.88 (−6.49 to −1.28)	0.003

* Adjusted for age, sex, and time on maintenance hemodialysis. RMST, restricted mean survival time; CI, confidence interval. RMST differences were calculated as sarcopenia minus no sarcopenia; negative values therefore indicate shorter restricted survival time among participants with sarcopenia. In the sensitivity Cox regression analysis adjusted for age, sex, and time on maintenance hemodialysis, sarcopenia was associated with a higher hazard of all-cause mortality (HR 2.62, 95% CI 1.06–6.45; *p* = 0.036; Table 5, Model 1). However, because the proportional-hazards assumption was not satisfied, this hazard ratio should not be interpreted as a constant relative effect throughout the 24-month follow-up period.

**Table 5:** Cox proportional-hazards sensitivity analyses of 24-month all-cause mortality (n = 176; 31 events).

Variable	Model 1: Adjusted HR (95% CI)	*p*	Model 2: Extended HR (95% CI)	*p*
Sarcopenia	2.62 (1.06–6.45)	0.036	2.89 (0.96–8.65)	0.058
Age, per year	1.03 (1.00–1.06)	0.070	1.03 (1.00–1.06)	0.043
Female sex	1.17 (0.57–2.40)	0.668	1.02 (0.50–2.09)	0.958
Time on maintenance HD, per year	0.84 (0.68–1.04)	0.117	0.81 (0.65–1.01)	0.058
Serum albumin, per 1 g/L	—	—	0.88 (0.80–0.98)	0.017
Serum phosphorus, per 1 mmol/L	—	—	2.20 (0.69–7.01)	0.183

CI, confidence interval; HD, hemodialysis; HR, hazard ratio. Model 1 was adjusted for sarcopenia, age, sex, and time on maintenance hemodialysis. Model 2 was additionally adjusted for serum albumin and serum phosphorus. Dashes indicate covariates not included in that model. Because the proportional-hazards assumption was not satisfied for sarcopenia, both models are reported as sensitivity analyses, and the hazard ratios are interpreted as average associations over the 24-month follow-up period rather than as constant relative hazards. The sarcopenia-by-sex interaction is reported in the text.

In an extended sensitivity Cox model additionally adjusted for serum albumin and serum phosphorus, the point estimate for sarcopenia remained elevated, although the association no longer reached conventional statistical significance (HR 2.89, 95% CI 0.96–8.65; *p* = 0.058; Table 5, Model 2). Older age (HR 1.03 per year, 95% CI 1.00–1.06; *p* = 0.043) and lower serum albumin (HR 0.88 per 1 g/L increase, 95% CI 0.80–0.98; *p* = 0.017) were associated with mortality. Serum phosphorus, sex, and time on maintenance hemodialysis were not independently associated with mortality. The attenuation of the sarcopenia estimate on addition of two further covariates to a model containing only 31 events is consistent with loss of precision rather than with disappearance of the association and is discussed in Section 4. Because the proportional-hazards assumption was not satisfied for sarcopenia, these Cox estimates were interpreted as sensitivity analyses rather than as constant relative hazards over follow-up.

There was no evidence that the association between sarcopenia and survival differed according to sex. The sarcopenia-by-sex interaction was not statistically significant in the adjusted RMST analysis (*p* = 0.964) or in the Cox sensitivity analysis (*p* = 0.641). In exploratory sex-stratified analyses, the log-rank *p*-value was 0.004 in men and 0.42 in women; these stratum-specific values do not by themselves indicate a difference between the sexes and are not interpreted as such.

Additional adjustment for Kt/V did not materially alter the findings. Sarcopenia remained associated with a shorter restricted survival time (adjusted RMST difference −3.76 months, 95% CI −6.32 to −1.20; *p* = 0.004). In the corresponding Cox sensitivity model, sarcopenia was associated with a higher hazard of all-cause mortality (HR 2.69, 95% CI 1.07–6.77; *p* = 0.035).

## 4. Discussion

In this single-center prospective cohort of patients receiving maintenance hemodialysis, confirmed sarcopenia defined according to EWGSOP2 criteria was present in approximately one quarter of participants and was associated with shorter restricted mean survival over 24 months after adjustment for age, sex, and time on maintenance hemodialysis. In the extended Cox sensitivity model that additionally included serum albumin and serum phosphorus, the point estimate for sarcopenia remained elevated, although the confidence interval widened and included unity. Given the small number of deaths and the additional parameters included in this model, the estimate is less precise and should be interpreted accordingly. At baseline, participants with sarcopenia were older and had lower serum albumin and phosphorus concentrations, lower dialysis adequacy, and longer time on maintenance hemodialysis. Because these variables were assessed concurrently with sarcopenia, they should be interpreted as cross-sectional correlates rather than as causes or temporal predictors.

The observed prevalence of 26.1% falls within the broad range reported in maintenance dialysis populations. Published estimates vary considerably, from approximately 4% to 63% [22], with many contemporary studies reporting values of roughly 18% to 40% [22,23,24]. Differences in diagnostic criteria, methods used to estimate muscle mass, and patient characteristics probably account for much of this variation. In an Australian single-center dialysis cohort, sarcopenia was assessed using EWGSOP-based criteria and bioimpedance-derived muscle mass [22]. Higher prevalence estimates were reported in a Korean cohort restricted to older patients with end-stage kidney disease [23], while sarcopenia was also common and associated with mortality in a Spanish cohort of older patients receiving hemodialysis [24]. The mean age in our cohort was 57.5 years, which was lower than in several of these studies. The observed prevalence therefore indicates that sarcopenia is clinically relevant even in a relatively younger maintenance hemodialysis population. Because our estimate was obtained using the strength-first EWGSOP2 algorithm together with bioimpedance-derived appendicular skeletal muscle index, it should not be considered interchangeable with estimates based on muscle mass alone or on other methods such as dual-energy X-ray absorptiometry [14]. It is also specific to the population and measurement approach used in this study. The inclusion of clinically stable patients with functioning arteriovenous fistulas who were able to complete functional testing may have resulted in some selection against frailer patients; prevalence in less selected hemodialysis populations could therefore differ and may be higher.

The association between sarcopenia and mortality is consistent with previous studies. Individual dialysis cohorts using muscle-mass-based or composite definitions have reported associations in the same direction, including a Chinese cohort in which sarcopenia was associated with poorer survival [25] and a Korean study reporting associations with both long-term mortality and cardiovascular events [26]. Systematic reviews and meta-analyses have reached similar conclusions across heterogeneous study populations and diagnostic approaches [15,16]. The present study extends this literature by applying the combined strength-and-mass EWGSOP2 definition in a South-eastern European hemodialysis population and by expressing the survival difference on an absolute time scale.

The prognostic contribution of the individual components of sarcopenia may, however, differ. Comparative analyses in dialysis populations have suggested that muscle strength may be more strongly associated with mortality than muscle mass, with low handgrip strength predicting mortality in settings where low muscle mass did not [27]. Because EWGSOP2 confirmation in our study required both components, their independent contributions cannot be separated. It therefore remains possible that muscle strength accounts for a larger proportion of the prognostic signal observed here. If so, assessment of muscle mass may contribute less additional prognostic information, although the present study was not designed to test this question. Whether thresholds derived from community-dwelling older adults are fully appropriate in patients with kidney failure also remains uncertain, given differences in body composition, uremic muscle dysfunction, and chronic fluid shifts [13].

The proportional-hazards assumption was not satisfied for sarcopenia, and RMST was therefore selected as the primary measure of the survival difference. RMST summarizes survival over a prespecified period without assuming that the relative hazard remains constant throughout follow-up and expresses the difference on an absolute time scale. In this cohort, sarcopenia was associated with approximately four fewer months of restricted mean survival during the 24-month observation period after adjustment for age, sex, and dialysis duration. This absolute measure may be easier to interpret clinically than a single hazard ratio when hazards are non-proportional.

Most previous studies of sarcopenia and mortality in dialysis populations have reported hazard ratios, while assessment of proportionality has not always been described. When proportional hazards do not hold, a single hazard ratio represents a summary across follow-up rather than a constant relative effect, which can complicate comparison between studies with different follow-up durations. In our study, the survival curves separated relatively early. This pattern may reflect differences in baseline health status between participants with and without sarcopenia, including differences in catabolic or comorbid burden. However, with only 31 deaths, the timing and shape of this divergence are estimated imprecisely and should not be interpreted mechanistically or as evidence for a specific intervention window.

Longer time on maintenance hemodialysis was associated with sarcopenia at baseline but was not independently associated with mortality in the survival models. One possibility is that sarcopenia partly reflects physiological changes accumulating during long-term dialysis and may therefore capture aspects of health status not represented by dialysis duration alone. The data cannot establish this interpretation, however. Survivor selection in a prevalent hemodialysis cohort may also attenuate the association between dialysis duration and mortality, and the present study cannot distinguish between these explanations.

Diabetes was not significantly associated with sarcopenia in this cohort (OR 1.56, 95% CI 0.71–3.45). This differs from the biological expectation that insulin resistance, impaired anabolic signaling, neuropathy, and reduced physical activity may contribute to muscle loss [6,7], as well as from reports of an association between diabetes and sarcopenia in hemodialysis populations [28]. The point estimate was above unity, but the confidence interval was wide, and only 36 participants had diabetes. The estimate therefore does not exclude a clinically relevant association. Measurement may also have contributed to this finding because body weight enters the Sergi equation positively, potentially influencing estimated appendicular muscle mass in heavier patients with type 2 diabetes. The extent of any such effect cannot be determined from our data. Selection of clinically stable patients who were able to complete handgrip testing may also have influenced the observed association. Thus, the absence of statistical significance in this cohort should not be interpreted as evidence that diabetes is unrelated to sarcopenia in patients receiving hemodialysis.

The association between lower Kt/V and sarcopenia also warrants cautious interpretation. Kt/V is normalized to the urea distribution volume, which is related to total body water and therefore to body composition. Differences in muscle mass may consequently influence Kt/V independently of a biological effect of dialysis dose on skeletal muscle. Confounding and reverse causation are also possible, as poor nutritional status, reduced muscle mass, and comorbidity may be related both to the muscle phenotype and to measures of dialysis adequacy. The present findings therefore do not imply that increasing dialysis dose would prevent or reverse sarcopenia; addressing that question would require interventional data.

Lower serum albumin was associated with both sarcopenia and mortality and was the only biochemical variable that remained independently associated with mortality in the extended Cox model. Although albumin is often used as a nutritional marker, it is also a negative acute-phase reactant and is influenced by inflammation, hydration status, comorbidity, protein losses, and hepatic synthesis. In the absence of a structured nutritional assessment, dietary intake data, or normalized protein catabolic rate, the association observed here should not be interpreted as direct evidence of malnutrition or protein-energy wasting [11]. The same limitation applies to inflammation: C-reactive protein was measured only once at baseline and therefore cannot characterize inflammatory burden over time. Composite tools such as the malnutrition-inflammation score [29] and the Controlling Nutritional Status score [30] could provide a broader assessment of nutritional risk, but the variables needed to calculate these scores were not available in the predefined dataset. Their relationship with EWGSOP2-defined sarcopenia would be useful to examine in future hemodialysis cohorts.

Serum phosphorus was inversely associated with sarcopenia. Although hyperphosphatemia is a familiar concern in kidney failure, lower phosphorus concentrations may also occur in patients with reduced dietary protein intake, because dietary protein and phosphorus intake are closely related. Lower phosphorus may also reflect phosphate-binder use, dietary restriction, reduced anabolic activity, or greater overall illness burden. In a multicenter cohort of 3552 hemodialysis patients followed for 24 months, hypophosphatemia was accompanied by lower serum albumin, lower protein intake, lower body mass index, and a lower lean tissue index and was associated with mortality after multivariable adjustment [31]. These findings are compatible with the possibility that low phosphorus may, in some settings, accompany reduced nutritional intake and lower lean tissue. This interpretation is also compatible with our cross-sectional finding, but our study did not assess dietary intake, phosphate-binder prescription, or adherence and did not reproduce an independent association between phosphorus and mortality. The present results therefore do not support any change in recommendations regarding phosphorus restriction.

Sarcopenia was slightly more prevalent in women than in men, but there was no evidence that sex modified its association with mortality. The sarcopenia-by-sex interaction was not statistically significant in either the adjusted RMST or Cox analysis. Although the sex-stratified Kaplan–Meier analysis was statistically significant in men but not in women, a difference in stratum-specific *p*-values does not by itself demonstrate effect modification. The small numbers of men and women with sarcopenia and the limited number of deaths further restrict the precision of these comparisons. Sex-specific associations between handgrip strength and health outcomes have been described in older populations [32], and EWGSOP2 itself uses sex-specific thresholds, but adequately powered studies would be required to determine whether the prognostic relevance of sarcopenia differs between men and women receiving hemodialysis. The present exploratory analyses do not support sex-specific conclusions.

This study has several limitations. Its observational design precludes causal inference, and the cross-sectional assessment of baseline correlates leaves open the possibility of reverse causation. Residual confounding is also likely because cardiovascular comorbidity, formal comorbidity indices, frailty, physical activity, dietary intake, normalized protein catabolic rate, hospitalization burden, and residual kidney function were not systematically recorded. These factors may be related to both sarcopenia and mortality. Only 31 deaths occurred. The primary survival model included four parameters, corresponding to approximately 7.8 events per parameter, whereas the extended sensitivity model included six parameters, corresponding to approximately 5.2 events per parameter. Estimates from the extended model, particularly the confidence intervals, should therefore be regarded as imprecise and potentially unstable. Several secondary and exploratory analyses were performed without adjustment for multiple comparisons, increasing the possibility of chance findings.

Physical performance was not assessed, preventing classification of severe sarcopenia and limiting characterization of the functional phenotype. Muscle mass was estimated using bioelectrical impedance and the Sergi equation, which was developed in free-living Caucasian older adults rather than in patients with ESKD. Altered hydration and tissue composition in kidney failure may introduce error relative to reference methods such as dual-energy X-ray absorptiometry or magnetic resonance imaging. Standardizing measurements to the immediate post-dialysis period of the mid-week session was intended to reduce, but cannot eliminate, the influence of fluid status. Cause-specific mortality was not adjudicated. Finally, this was a single-center study restricted to clinically stable patients able to complete the required assessments, which limits generalizability to the broader hemodialysis population.

## 5. Conclusions

In this single-center prospective cohort, confirmed sarcopenia defined according to EWGSOP2 criteria was present in approximately one quarter of patients receiving maintenance hemodialysis and was associated with approximately four months shorter restricted mean survival over 24 months after adjustment for age, sex, and time on maintenance hemodialysis. Older age, lower serum albumin and phosphorus concentrations, lower dialysis adequacy, and longer time on hemodialysis were associated with sarcopenia at baseline, although the cross-sectional nature of these associations precludes causal interpretation. There was no evidence that the association between sarcopenia and survival differed by sex. These findings suggest that EWGSOP2-defined sarcopenia provides prognostic information in patients receiving maintenance hemodialysis, but they do not establish that routine screening or interventions based on sarcopenia status improve clinical outcomes. Larger prospective studies and interventional trials are needed to determine whether identification and targeted management of sarcopenia can translate into improved outcomes.

## Figures and Tables

**Table 1:** Baseline characteristics of the study population, overall and by sex.

Characteristic	Total (n = 176)	Men (n = 106)	Women (n = 70)
Age, years	57.5 ± 13.0	57.4 ± 13.2	57.7 ± 12.7
**Primary cause of ESKD, n (%)**			
Diabetic kidney disease	36 (20.5)	21 (19.8)	15 (21.4)
Hypertension	53 (30.1)	40 (37.7)	13 (18.6)
Glomerulonephritis	16 (9.1)	10 (9.4)	6 (8.6)
Polycystic kidney disease	25 (14.2)	16 (15.1)	9 (12.9)
Other or unknown	46 (26.1)	19 (17.9)	27 (38.6)
Time on maintenance hemodialysis, years	5.7 ± 2.0	5.5 ± 1.9	5.9 ± 2.2
Kt/V	1.33 ± 0.30	1.31 ± 0.32	1.35 ± 0.27
Weight, kg	66.6 ± 15.4	73.0 ± 12.4	56.8 ± 14.3
Height, m	1.66 ± 0.08	1.69 ± 0.07	1.61 ± 0.06
Body mass index, kg/m^2^	25.0 ± 5.6	25.2 ± 5.9	24.6 ± 5.1
Waist circumference, cm	91.4 ± 11.1	93.8 ± 10.7	87.7 ± 10.8
Body fat, %	28.5 ± 8.7	24.8 ± 7.7	34.1 ± 7.1
ASMI, kg/m^2^	6.8 ± 1.3	7.3 ± 1.1	6.0 ± 1.1
Handgrip strength, kg	22.4 ± 7.4	24.6 ± 8.1	19.0 ± 4.4

Data are mean ± standard deviation, median [interquartile range], or number (percentage). Percentages are column percentages within each sex group. No hypothesis tests were performed for between-sex comparisons. ASMI, appendicular skeletal muscle index; ESKD, end-stage kidney disease; Kt/V, single-pool dialysis adequacy index.

## Data Availability

The data supporting the findings of this study are available from the corresponding author upon reasonable request. The data are not publicly available because they contain information that could compromise the privacy of research participants.
